# Socioeconomic Status and Students’ Mental Health during the COVID-19 University Closure: Mediating Roles of Perceived Social Support and Self-Efficacy

**DOI:** 10.3390/bs13100871

**Published:** 2023-10-23

**Authors:** Liang Huang, Dongsheng Wang

**Affiliations:** 1 Department of Public Administration, Southeast University, Nanjing 211189, China; 2 Faculty of Education, Northwest Normal University, Lanzhou 730070, China

**Keywords:** SES, perceived social support, self-efficacy, mental health

## Abstract

Despite the need for urgent actions in response to the exacerbated inequalities in mental health resulting from the COVID-19 pandemic, there remains a significant gap in research into the relationships and underlying mechanisms between socioeconomic status (SES) and various mental health outcomes among students during the COVID-19 university closure. With a sample of 839 students from a university in Lanzhou, the capital city of China’s Gansu Province, which was closed during the 2022 autumn semester due to the COVID-19 outbreak, this study examined the relationships between SES and both the negative and positive mental health outcomes, with a particular inquiry into the mediating roles of perceived social support and self-efficacy. The results show that SES had significant and negative total associations with psychological distress (β = −0.119, *p* < 0.001) and loneliness (β = −0.132, *p* < 0.001), while having significant and positive total associations with life satisfaction (β = 0.90, *p* < 0.01) and affective well-being (β = 0.108, *p* < 0.01). Moreover, perceived social support and self-efficacy independently and sequentially mediated the associations between SES and various mental health outcomes. Research implications for the design and improvement of university measures to reduce the socioeconomic inequalities in students’ mental health are also discussed.

## 1. Introduction

The widespread coronavirus disease 2019 (COVID-19) pandemic and the ensuing uncertainty, university closure, and routine disruption have created severe hindrances to the mental health of university students in countries across the world [1,2]. This can be particularly so for socioeconomically disadvantaged students who might otherwise have benefited from university services and support systems as a means of risk avoidance, adaptive coping, and mental health maintenance [3,4]. Empirically, multiple studies have revealed disproportionate impacts of the pandemic on various mental health outcomes between university students from different socioeconomic groups, resulting in potentially enduring repercussions over time [5,6]. Despite the need for urgent actions in response to the exacerbated inequalities in mental health resulting from the COVID-19 pandemic [7], there remains a lack of research into the relationships and underlying mechanisms between socioeconomic status (SES) and various mental health outcomes among students during the COVID-19 university closure. This significant research gap would impede the design of targeted policies and the implementation of counteracting interventions.

It is exactly this research gap that we attempted to address in the present study. Specifically, we examined the relationships between SES and both the negative and positive mental health outcomes during the COVID-19 university closure, with a particular focus on the mediating roles of perceived social support and self-efficacy. This is due to that individuals from different socioeconomic backgrounds have varying levels of perceived social support [8] and internal efficacy beliefs [9], which contribute to multi-layered coping systems that interact to influence various mental health outcomes [10,11]. This holds particularly true for university students, as they are tasked with exploring possibilities within the social world and building self-beliefs towards goals of life [12]. Indeed, research into the impacts of pandemic-induced crises has explicated their importance in influencing individuals’ ability to cope and maintain well-being [13,14]. Thus, these factors can be crucial in understanding the disparities in various mental health outcomes across different socioeconomic conditions [15]. To address the noticeable research gap and to contribute to intervention measures that help mitigate the socioeconomic inequalities in mental health, the present study was conducted with a sample of Chinese students experiencing university closure during the COVID-19 pandemic.

## 2. Literature Review

### 2.1. Socioeconomic Status (SES) and Students’ Mental Health during the COVID-19 University Closure

Defined as one’s social position compared to others in terms of access to or control over favorable resources for development and functioning, SES is usually calculated as a combined total measure of household wealth, parental educational attainment, and parental occupational status [16]. Over decades, empirical evidence has accumulated and identified SES as one of the most prominent social and environmental determinants of mental health and well-being [8]. As informed by the fundamental cause theory [17], individuals with higher SES are more capable of flexibly mobilizing and using resources, including money, power, social networks, and knowledge, towards risk avoidance and adaptive coping than those of lower SES [18], thus maintaining advantages of health and well-being [17]. Evidence has shown that SES is significantly associated with students’ mental health, with higher-SES students at a lower risk of having psychological distress symptoms (e.g., anxiety, depression, and stress) and loneliness, compared to lower-SES students [19]. Increasing research has also investigated the SES impacts on positive mental health outcomes of life satisfaction and affective well-being [20,21].

Mental health, as described by the World Health Organization (WHO), represents a well-being state that enables individuals to unlock their potential, successfully navigate life and work challenges, and contribute to societal benefits at large [22]. As a multifaceted concept, it involves not only the mere absence of negative mental health outcomes but also the presence of positive mental states [22]. The COVID-19 pandemic and the ensuing closure of universities represent an atypical experience that is anticipated to worsen the protracted socioeconomic disparities in the mental health of university students [17,23]. For instance, evidence shows that due to the university shutdown and accompanying social isolation among staff and students, students living in socioeconomically disadvantaged conditions, who had already faced higher risks of mental problems, were cut off from previous beneficial bonds and thus were more prone to elevated pathological distress, loneliness, and diminished affective well-being and satisfaction with life [5,24]. Despite the deepened SES influences during the COVID-19 university closure [4], scant studies have elucidated the relationships between SES and both negative and positive mental health outcomes. To address the research gap, the present study hypothesizes that:

**Hypothesis** **1.** *SES had negative associations with negative mental health outcomes, including psychological distress (H1a) and loneliness (H1b), while having positive associations with positive mental health outcomes of life satisfaction (H1c) and affective well-being (H1d) among students during the COVID-19 university closure.*

### 2.2. Mediating Role of Perceived Social Support

Perceived social support refers to subjective perceptions regarding the availability and adequacy of various resources or assistance that individuals think they can mobilize when in need [25]. Despite the multifarious ways that perceived social support influences mental health, researchers have consistently identified it as practically useful in cushioning negative stress attributes and improving mental health [26]. This role can be more prominent in times of adversity and hardship when individuals’ beliefs regarding the availability of influential social resources to cope with stress for mental health become irreplaceably important [27]. This is particularly true for university students struggling to adapt themselves to stressful young adulthood by taking advantage of the proximate opportunities and support within their social networks and interpersonal relationships [28]. In response to escalated pathological symptoms of distress and loneliness, diminished life satisfaction, and reduced affect specific to the COVID-19 university closure and the changes it brought about to university students [29,30], substantial research has elucidated the importance of perceived support from family, peers, and teachers in addressing such mental health challenges [14,31].

Despite the salient importance, the availability and adequacy of perceived social support have been found to vary greatly across different socioeconomic groups [8,32]. It has been evidenced that higher-SES individuals usually perceive higher levels of financial support, positive parenting, supportive friendships, and social integration, thus reporting higher levels of life satisfaction and positive mood states [33]. In contrast, lower-SES individuals are more vulnerable to poorer mental health outcomes, including psychological distress, loneliness, and low life satisfaction, due to lower perceived supportive resources that can be garnered for stress coping [34,35]. The unequal distribution of perceived social support as well as its empirical relevance to various mental health outcomes has led scholars to call for the use of digital resources and tools to support low-SES individuals in staying socially connected and integrated, so as to counteract pandemic isolation and retain mental health and well-being [36,37]. As such, there is sufficient reason to assert that:

**Hypothesis** **2.** *Perceived social support significantly mediated the associations between SES and the negative mental health outcomes of psychological distress (H2a) and loneliness (H2b), as well as the positive mental health outcomes of life satisfaction (H2c) and affective well-being (H2d).*

### 2.3. Mediating Role of Self-Efficacy

Self-efficacy, defined as one’s perceived beliefs in their own ability to effectively manage and perform functioning towards desirable outcomes [38], represents one of the most prominent psychological coping factors that determine individual development and well-being [39]. Research has revealed that a high level of efficacy beliefs fosters active stress management, emotional control, task engagement, resilience, and optimism, thus allaying pervasive distress symptoms among university students and enhancing their cognitive responses to and emotional feelings towards life [40,41,42]. In contrast, a low self-efficacy level has been found related to maladaptive coping choices, dysfunctional emotional regulation, social avoidance, self-doubt, and pathological loneliness, all of which take a heavy toll on mental health [11,43]. This is especially the case when university students are transitioning into the stage of developing identity and beliefs through managing instabilities and functioning [44]. Due to extensive social isolation and university closure during COVID-19, self-efficacy can be particularly relevant as students, in the presence of diverse life stresses and demands, find themselves responsible for tackling long-term stress, psychological distress, loneliness, and reduced affective well-being [45].

It is evidenced that self-efficacy as an internal psychological process targeting mental health problems and the deterioration of positive well-being is deeply affected by external socioeconomic conditions [46]. As informed by the theoretical sources of self-efficacy [38], SES can influence self-efficacy through multiple information channels [47], thereby exerting knock-on effects on mental health. For instance, higher-SES individuals who have better control over material and financial resources may have more mastery experiences of succeeding in coping with adversities and stress than those who do not [7]. Furthermore, higher SES helps individuals garner more useful knowledge and vicarious experiences that allow appropriate approaches to stressful events [48]. Moreover, higher-SES students can develop stronger self-efficacy through perceiving more social persuasion information (e.g., trust, praise, and encouragement) from significant others than lower-SES students [35]. Supporting this idea, Gecas [49], as well as the more recent work by Wiederkehr, Darnon, Chazal, Guimond, and Martinot [47], argues that students with different SES have varying control over life conditions, access to favorable information, and positions in the social hierarchy, which can impact their self-efficacy development regarding fitting into the social environment and sustaining mental health. In this sense, it can be hypothesized that:

**Hypothesis** **3.** *Self-efficacy significantly mediated the associations between SES and the negative mental health outcomes of psychological distress (H3a) and loneliness (H3b), as well as the positive mental health outcomes of life satisfaction (H3c) and affective well-being (H3d).*

### 2.4. Sequentially Mediating Roles of Perceived Social Support and Self-Efficacy

Research has revealed a positive relationship between perceived social support and self-efficacy [46,50]. That is, one’s efficacy beliefs in performing functioning and addressing adversities can be sourced and nurtured when perceiving support from significant others [51]. For university students struggling against heightened mental stress, their staying connected to family, friends, and teachers and perceiving various support are helpful not only to generate mastery and vicarious experiences of coping but also to stimulate constructive information of social persuasion, all of which foster self-efficacy [38,52]. Research has identified perceived social support and self-efficacy as useful social and psychological coping resources that coalesce to enable optimized functioning and bolster mental health and well-being [11,50]. For instance, Karademas [11] revealed combined effects of self-efficacy and social support on life satisfaction and depressive symptoms. Rippon, Shepherd, Wakefield, Lee, and Pollet [13] found functional social support interacted with self-efficacy to influence psychological well-being. Similarly, Huang and Zhang [14] found that perceived social support influenced college students’ positive mental health via various psychological resources, including efficacy. Despite the apparent relevance of these two psychosocial variables to the inequalities in mental health between different socioeconomic groups during the COVID-19 pandemic [7], no studies, to the best of our knowledge, have examined the effects of SES on both the negative and positive mental health outcomes via perceived social support and self-efficacy. Nonetheless, given that both perceived social support and self-efficacy are socioeconomically patterned and function synergistically to shape abilities in stress coping, emotional regulation, and alleviation of pandemic-induced distress and loneliness [13], the following hypothesis can be proposed:

**Hypothesis** **4.** *Perceived social support and self-efficacy sequentially mediated the associations between SES and the negative mental health outcomes of psychological distress (H4a) and loneliness (H4b), as well as the positive mental health outcomes of life satisfaction (4c) and affective well-being (H4d).*

## 3. Methods

### 3.1. Data and Sample

The study is part of a project on the life of university students during the COVID-19 pandemic in China. A convenience sampling procedure was used to sample full-time undergraduate students from an urban university in Lanzhou, the capital city of China’s Gansu Province, which was closed during the 2022 autumn semester as an emergency response to the COVID-19 outbreak. Specifically, an online questionnaire was generated and disseminated by the researchers to accessible teachers and students for convenience. They were then asked to further distribute the questionnaire to students within their social networks. A total of 839 valid responses were identified from a diverse group of university students with varying characteristics, such as gender, grade, and major. Of the participants, there were 649 (77.4%) females and 190 (22.6%) males. As for the grade, 510 (60.8%) students were in the first year, 206 (24.6%) were in the second year, and 123 (14.7%) were in the third year or more. There were 188 (22.4%) students majoring in sciences and engineering, 523 (62.3%) majoring in humanities and social sciences, and 128 (15.3%) majoring in arts and sports.

### 3.2. Variables

#### 3.2.1. Mental Health

Mental health was measured through a combination of negative and positive outcomes. As for negative mental health outcomes, the 6-item Kessler Psychological Distress Scale (K6) [53] and the 8-term UCLA Loneliness Scale (ULS-8) [54] were utilized, respectively, to measure psychological distress (e.g., nervous, restless or fidgety, and worthless; Cronbach α = 0.938, in the present study) and feelings of loneliness (e.g., lacking companionship, feeling left out, and feeling isolation from others; Cronbach α = 0.768, in the present study) with 5-point Likert scales that ranged from 1 (none of the time) to 5 (all of the time). As for positive mental health outcomes, a single-item scale that ranged from 0 (very dissatisfied) to 10 (very satisfied) was used to measure students’ satisfaction with life during university closure. Moreover, the WHO-5 well-being index used in the Survey on Social and Emotional Skills [55] with a range of 1 (none of the time) to 5 (all of the time) was used to measure students’ affective well-being (e.g., “I have felt active and vigorous”; Cronbach α = 0.952, in the present study).

#### 3.2.2. Self-Efficacy

Self-efficacy was assessed through the 5-item Self-efficacy Scale [56], a 4-point Likert scale with a range of 1 (strongly disagree) to 4 (strongly agree). The scale was used to measure students’ beliefs in their competence in performing and managing functioning to accomplish desired goals during the COVID-19 university closure. The calculated Cronbach α (0.890) in the present study showed good reliability.

#### 3.2.3. Perceived Social Support

Students’ perceptions of social support were measured based on Zimet et al. [57], with a 6-point Likert scale ranging from 1 (strongly disagree) to 6 (strongly agree). The 12-item scale was used to assess three sources of available support (i.e., family, friends, and significant others) as reported by students experiencing university closure. The estimated Cronbach’s α was 0.924, indicating good reliability.

#### 3.2.4. Socioeconomic Status

SES was measured based on the SES scale used in the Program for International Student Assessment (PISA) [58], which was calculated as a combined and average score of three standardized variables: the highest parental education in years of schooling in line with the International Standard Classification of Education (ISCED 2011); the highest parental occupational status based on the International Standard Classification of Occupations (ISCO-08); and family wealth as measured by four family affluence items (i.e., own bedroom, family car, number of computers, and vacations prior to the pandemic) [59]. The Cronbach’s α (0.781) for the SES scale showed good internal consistency.

#### 3.2.5. Control Variables

Students’ gender, grade, and major were controlled to exclude the impact of potential confounding variables [60,61].

### 3.3. Data Analysis

Mplus 7.0 software was used to analyze data and test hypotheses. First, a correlation analysis was preliminarily conducted to examine correlations. Then a path analysis model was constructed with gender, grade, and major being controlled to analyze the relationships between SES and mental health variables (i.e., psychological distress, loneliness, life satisfaction, and affective well-being) as well as to analyze the mediating roles of perceived social support and self-efficacy. Based on the path analysis model, the bias-corrected bootstrapping method with 2000 resamplings was used to conduct inference for mediation analysis. This analytical strategy can generate accurate standard errors for the point estimates of the 95% confidence intervals (CIs) regarding the mediating effects [62]. Before the data analysis, normal distribution tests were conducted for all instruments, with the calculated absolute values of both skewness and kurtosis being less than 3.

## 4. Results

### 4.1. Correlation Analysis

As Table 1 shows, there were significant correlations among the four mental health outcomes. As anticipated, SES had negative correlations with negative indicators of psychological distress (γ = −0.125, *p* < 0.001) and loneliness (γ = −0.131, *p* < 0.001), while having positive correlations with positive outcomes of life satisfaction (γ = 0.091, *p* < 0.01) and affective well-being (γ = 0.109, *p* < 0.01). Moreover, both perceived social support and self-efficacy had negative correlations with the two negative mental health outcomes, while having positive correlations with the two positive mental health indicators. In addition, SES had positive correlations with both perceived social support (γ = 0.178, *p* < 0.001) and self-efficacy (γ = 0.206, *p* < 0.001). Perceived social support had positive correlations with self-efficacy (γ = 0.419, *p* < 0.001).

### 4.2. Path Analysis Model

**Figure 1:**
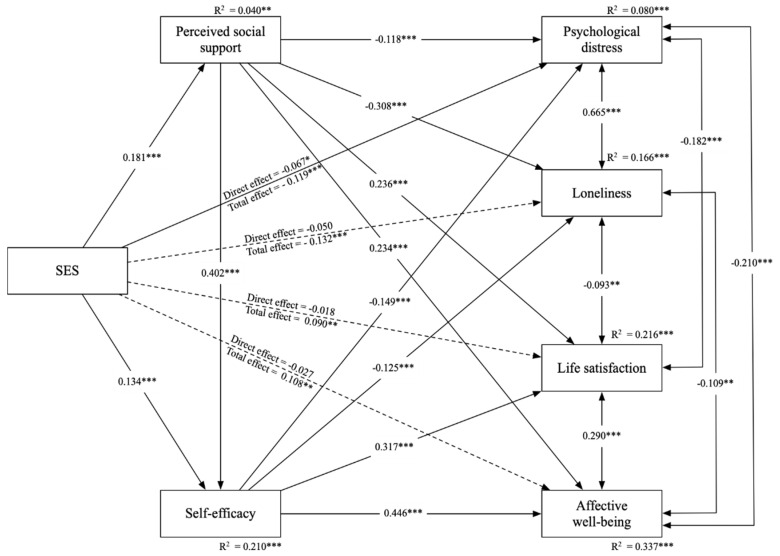
Results of path analysis model. Note: Standardized results are demonstrated. Significant paths are denoted by solid lines and nonsignificant ones by dotted lines. Control variables (i.e., gender, grade, and major) were controlled for all the paths among focus variables. * *p* < 0.05, ** *p* < 0.01, *** *p* < 0.001.

Figure 1 shows the results of the path analysis model. With gender, grade, and major being controlled for all the paths among focus variables, the path analysis model accounted for 8.0%, 16.6%, 21.6%, and 33.7% of the variance in psychological distress, loneliness, life satisfaction, and affective well-being, denoting a relatively high explanatory power for affective well-being, life satisfaction, and loneliness and a relatively low one for psychological distress. As illustrated in Figure 1, without the inclusion of the mediators of perceived social support and self-efficacy, SES had significant and negative total effects on negative mental health outcomes of psychological distress (β = −0.119, *p* < 0.001) and loneliness (β = −0.132, *p* < 0.001), while having significant and positive total effects on positive mental health outcomes of life satisfaction (β = 0.90, *p* < 0.01) and affective well-being (β = 0.108, *p* < 0.01), thus supporting H1a, H1b, H1c, and H1d.

Figure 1 also shows that with the inclusion of the mediators of perceived social support and self-efficacy, SES had a significant and direct relationship with psychological distress (β = −0.067, *p* < 0.05) but had no significant or direct relationship with other mental health outcomes. Moreover, SES had a significant and positive relationship with perceived social support (β = 0.181, *p* < 0.001) and self-efficacy (β = 0.134, *p* < 0.001), respectively. Perceived social support had a significant and positive relationship with self-efficacy (β = 0.402, *p* < 0.001), life satisfaction (β = 0.236, *p* < 0.001), and affective well-being (β = 0.234, *p* < 0.001), respectively, and had a significant and negative relationship with psychological distress (β = −0.118, *p* < 0.001) and loneliness (β = −0.308, *p* < 0.001), respectively. Moreover, self-efficacy had a significant and positive relationship with life satisfaction (β = 0.317, *p* < 0.001) and affective well-being (β = 0.446, *p* < 0.001), respectively, and had a significant and negative relationship with psychological distress (β = −0.149, *p* < 0.001) and loneliness (β = −0.125, *p* < 0.001).

Table 2 presents the results of mediation analysis using the bootstrapping method. The results reveal that perceived social support significantly mediated the associations between SES and the negative mental health outcomes of psychological distress (β = −0.021, *p* < 0.01, 95% Bootstrap CIs: −0.040 to −0.008) and loneliness (β = −0.056 *p* < 0.001, 95% Bootstrap CIs: −0.085 to −0.034), as well as the positive mental health outcomes of life satisfaction (β = 0.043, *p* < 0.001, 95% Bootstrap CIs: 0.025 to 0.068) and affective well-being (β = 0.043, *p* < 0.001, 95% Bootstrap CIs: 0.025 to 0.064). Thus, research hypotheses H2a, H2b, H2c, and H2d were supported. Moreover, self-efficacy significantly mediated the associations between SES and psychological distress (β = −0.020, *p* < 0.05, 95% Bootstrap CIs: −0.039 to −0.007), loneliness (β = −0.017, *p* < 0.05, 95% Bootstrap CIs: −0.035 to −0.005), life satisfaction (β = 0.043, *p* < 0.01, 95% Bootstrap CIs: 0.019 to 0.069), and affective well-being (β = 0.060, *p* <0.001, 95% Bootstrap CIs: 0.031 to 0.092), supporting research hypotheses H3a, H3b, H3c, and H3d. In addition, perceived social support and self-efficacy sequentially mediated the associations between SES and psychological distress (β = −0.011, *p* < 0.01, 95% Bootstrap CIs: −0.022 to −0.004), loneliness (β = −0.009, *p* < 0.01, 95% Bootstrap CIs: −0.018 to −0.003), life satisfaction (β = 0.023, *p* < 0.001, 95% Bootstrap CIs: 0.015 to 0.036), and affective well-being (β = 0.033, *p* < 0.001, 95% Bootstrap CIs: 0.019 to 0.049), respectively. Hence, research hypotheses H4a, H4b, H4c, and H4d were supported.

## 5. Discussion

The COVID-19 pandemic and resulting uncertainty, university shutdown, and routine disruption have posed severe hindrances to students’ mental health in multiple ways [1,2], particularly for vulnerable low-SES individuals [3]. Despite the need for urgent actions to mitigate disproportionate pandemic impacts and exacerbated socioeconomic inequalities in the mental health [7], there is a lack of research into the relationships and underlying mechanisms between SES and various mental health outcomes during the COVID-19 university closure. To fill the research gap, this study examined the relationships between SES and both the negative and positive mental health outcomes during the COVID-19 university closure, with an inquiry into the mechanisms of perceived social support and self-efficacy.

It was discovered that SES exerted negative total relationships with negative mental health indicators, including psychological distress and loneliness, while having positive total associations with positive mental health outcomes of life satisfaction and affective well-being. In line with the fundamental cause theory [17] and studies that found university students to rely heavily on flexible socioeconomic resources to mitigate risks, adapt effectively, and sustain mental health and well-being [4,24], the results substantiate that students with different SES exhibit significant variance in their approach to mental health [42], a multifaceted concept that involves both the absence of negative mental symptomology and the presence of positive well-being [22]. Indeed, researchers have highlighted the heightened influences of SES on various mental health outcomes during the COVID-19 university closure [5,63]. In the face of the shutdown of university services and social isolation, low-SES students lacked economic stability, flexible resources, and access to additional mental health support systems needed to combat elevated psychological distress, feelings of loneliness, and the deterioration of affective well-being [17]. This vulnerability would make them more susceptible to these overwhelming life challenges and stresses than high-SES students.

Furthermore, perceived social support was found to mediate the associations between SES and various mental health outcomes. On the one hand, the results are in line with previous studies denoting that students with a strong sense of support, care, and connections to important individuals would experience lower psychological distress, loneliness, and higher levels of life satisfaction and positive affect compared to those lacking support [30]. On the other hand, the results corroborate differentiated availability and adequacy of perceived social support between students from different SES groups [32]. While high-SES students had more perceived support from both their families and external networks to address heightened negative symptomology and maintain positive well-being [4,33], low-SES students in resource-constrained settings cannot be furnished with necessary social support to mitigate the common pathological symptoms of distress and loneliness and stave off loss in positive well-being [34,36]. The research findings contribute to the literature by identifying perceived social support as an external social mechanism that accounts for the socioeconomic inequalities in multiple mental health outcomes during the COVID-19 university closure [31].

In addition, self-efficacy was also found to mediate the associations between SES and various mental health outcomes, which is consistent with previous studies highlighting the role of self-efficacy in internalizing socioeconomic information and influencing functioning management and mental well-being [43,46]. On the one hand, students with different SES had unequal access to or control over material, financial, social, and knowledge resources for coping [7], resulting in diverse mastery and vicarious experiences and social persuasion information in relation to efficacious beliefs towards managing stressful events and adapting to the external environment [38,47]. On the other hand, higher-SES students with a stronger sense of efficacy would be driven by their internal beliefs to employ active and adaptive strategies when faced with the overwhelming stresses arising from the COVID-19 university closure, thereby leading to lower levels of psychological distress and loneliness feelings [40,64]. In contrast, lower-SES students equipped with weaker self-efficacy cannot adapt well to stressful life and therefore experience heightened negative symptomology and deteriorated affective well-being [11]. As such, self-efficacy was identified in this study as an internal psychological mechanism through which SES influenced both negative and positive mental health outcomes.

Moreover, perceived social support and self-efficacy acted as sequential mediators in the associations between SES and multiple mental health outcomes. In other words, higher-SES students during the COVID-19 university closure can perceive more social support, which results in greater self-efficacy and, ultimately, better mental health compared to lower-SES students. The results corroborate that for university students suffering severe mental stress, their perception of support from significant others can be prominent to the development of internal self-efficacy [52]. The results also resonate with studies emphasizing the combined effects of perceived social support and self-efficacy as important coping resources on psychological functioning and multiple mental health outcomes [11,14,50]. These effects were more pronounced for those navigating times of adversity during the COVID-19 university closure [13]. Moreover, the results reveal that the SES effects on various mental health outcomes can be transmitted through perceived social support and self-efficacy in sequence, thus unravelling the multi-layered and interactive nature of these social and psychological resources in accounting for the disparities in stress coping and mental health maintenance among individuals from diverse socioeconomic backgrounds [7,15]. Given that no studies to date have elucidated how perceived social support and self-efficacy interact to explain the socioeconomic inequalities in different mental health outcomes, this study advances the literature by unpacking a sequentially mediating mechanism between SES and both the negative and positive mental health outcomes among students during the COVID-19 university closure.

## 6. Implications

The results of this study provide some practical implications. First, this study found significant associations between SES and both negative and positive mental health outcomes during the COVID-19 university closure. Although countries around the world have now relaxed the pandemic restrictions to push higher education institutions back to the normal routine [65], the evolution of coronavirus and its disruptions continue to disproportionally affect the mental health of vulnerable low-SES students [66]. Therefore, universities in times of crisis should seek to develop whole-campus measures, including providing emergency financial aid, streamlining helpline and online counseling services, and fostering an inclusive and supportive campus culture, to safeguard the mental health of all students regardless of their socioeconomic backgrounds [67].

Moreover, this study identified perceived social support as a critical social resource accounting for the associations between SES and various mental health outcomes. In practice, university mental health professionals should provide tailored services and target support for socioeconomically disadvantaged students to reduce the inequalities in mental health. Universities may also proactively build partnerships with families, student unions, and social organizations to enhance the availability and adequacy of multiple supportive resources for vulnerable low-SES students. They may also help students reduce the stigma surrounding mental health and encourage students to seek help when they experience heightened psychological distress, loneliness, and a decline in positive affective well-being, especially during large-scale crises.

In addition, this study identified self-efficacy as an important psychological mechanism connecting SES and various mental health outcomes. To support low-SES students in strengthening their efficacy beliefs in managing pervasive psychological distress symptoms, loneliness, and the disproportionate loss of positive affect and life satisfaction during times of great threat, university managers may implement selective interventions, such as online group training or individual counseling. These interventions should encompass content that focuses on fostering students’ self-confidence in dealing with stress appropriately and effectively addressing various mental health issues [68].

Moreover, this study also found that perceived social support and self-efficacy sequentially mediated the associations between SES and both negative and positive mental health outcomes. Hence, when designing and implementing intervention measures to mitigate the exacerbated and prolonged socioeconomic inequalities in mental health caused by the pandemic, university managers and mental health professionals should simultaneously attend to the availability and adequacy of external social support and the improvement of internal efficacy beliefs among students suffering from socioeconomic vulnerabilities. For instance, social support systems incorporating activities or programs that foster mastery (e.g., stress management courses) or vicarious (e.g., video testimonials) experiences of successful functioning as well as providing positive social persuasion information should be developed to help these students build confidence in addressing stressful situations and ameliorating mental health and well-being [41].

## 7. Conclusions and Limitations

This study advances empirical understanding as to the relationships as well as the underlying mechanisms between SES and various mental health outcomes among students during the COVID-19 university closure. In line with previous studies denoting that individuals heavily dependent on socioeconomic resources to manage pandemic-related stress and maintain mental health and well-being [4,7], this study found significant associations between SES and both the negative (i.e., psychological distress and loneliness) and positive (i.e., life satisfaction and affective well-being) mental health outcomes. Particularly, this study advances the literature by identifying the mediating roles of perceived social support and self-efficacy with respect to these associations.

Despite the research’s significance, several limitations exist. First, nonprobability convenience sampling was employed to select participants from a university in Lanzhou, Gansu Province, China, limiting the representativeness and the generalizability of the research findings. Nonetheless, the relationships identified in this study can shed an important light on studies within similar contexts. More studies that use large-scale and representative data should be conducted to validate the findings. Second, cross-sectional data were used in this study, which cannot warrant causalities but only associations. As such, future studies are suggested to utilize longitudinal data to draw causal inferences. Finally, perceived social support was conceptualized as a combined construct in this study, and future research is required to explore how individual sources of perceived support (e.g., family, friends, or teachers) mediate the associations between SES and various mental health outcomes.

## Figures and Tables

**Table 1:** Results of correlation analysis.

	1	2	3	4	5	6	7
1. Psychological distress	1						
2. Loneliness	0.684 ***	1					
3. Life satisfaction	−0.264 ***	−0.243 ***	1				
4. Affective well-being	−0.295 ***	−0.276 ***	0.476 ***	1			
5. Self-efficacy	−0.204 ***	−0.253 ***	0.411 ***	0.541 ***	1		
6. Perceived social support	−0.195 ***	−0.375 ***	0.365 ***	0.415 ***	0.419 ***	1	
7. SES	−0.125 ***	−0.131 ***	0.091 **	0.109 **	0.206 ***	0.178 ***	1
Mean	2.022	2.210	6.775	3.129	3.023	4.709	0.000
SD	0.774	0.603	2.030	0.893	0.479	0.810	0.834

Note: Standardized coefficients are reported. ** *p* < 0.01, *** *p* <0.001.

**Table 2:** Results of mediation analysis.

	β	S.E.	95% Bootstrap CIs
Dependent variable: Psychological distress			
SES → Perceived social support → Psychological distress	−0.021 **	0.008	[−0.040, −0.008]
SES → Self-efficacy → Psychological distress	−0.020 *	0.008	[−0.039, −0.007]
SES → Perceived Social support → Self-efficacy → Psychological distress	−0.011 **	0.004	[−0.022, −0.004]
Dependent variable: Loneliness			
SES → Perceived social support → Loneliness	−0.056 ***	0.013	[−0.085, −0.034]
SES → Self-efficacy → Loneliness	−0.017 *	0.008	[−0.035, −0.005]
SES → Perceived Social support → Self-efficacy → Loneliness	−0.009 **	0.004	[−0.018, −0.003]
Dependent variable: Life satisfaction			
SES → Perceived Social support → Life satisfaction	0.043 ***	0.011	[0.025, 0.068]
SES → Self-efficacy → Life satisfaction	0.043 **	0.013	[0.019, 0.069]
SES→ Perceived Social support→ Self-efficacy → Life satisfaction	0.023 ***	0.005	[0.015, 0.036]
Dependent variable: Affective well-being			
SES→ Perceived Social support → Affective well-being	0.043 ***	0.010	[0.025, 0.064]
SES→ Self-efficacy → Affective well-being	0.060 ***	0.015	[0.031, 0.092]
SES→ Perceived Social support → Self-efficacy → Affective well-being	0.033 ***	0.008	[0.019, 0.049]

Note: Standardized results are demonstrated. * *p* < 0.05, ** *p* <0.01, *** *p* <0.001.

## Data Availability

The data presented in this study are available on request from the corresponding author.
